# Proteome analysis identified proteins associated with mitochondrial function and inflammation activation crucially regulating the pathogenesis of fatty liver disease

**DOI:** 10.1186/s12864-021-07950-2

**Published:** 2021-09-04

**Authors:** Letian Zhang, Tingjun Liu, Chengzhang Hu, Xuan Zhang, Qin Zhang, Kerong Shi

**Affiliations:** Shandong Key Laboratory of Animal Bioengineering and Disease Prevention, College of Animal Science and Technology, Shandong Agricultural University, Taian, Shandong 271018 People’s Republic of China

**Keywords:** Nonalcoholic fatty liver disease (NAFLD), Mitochondrial dysfunction, Inflammation activation, Metabolic disorder, Animal model

## Abstract

**Background:**

Fatty liver disease prevalently occurs in commercial postpartum dairies, resulting in a worldwide high culling rate because of their subsequent limitations of production and reproduction performance.

**Results:**

Fatty liver-specific proteome and acetylome analysis revealed that energy metabolism suppression closely associated with mitochondrial dysfunction and inflammation activation were shown to be remarkable biological processes underlying the development of fatty liver disease, furthermore, acetylation modification of proteins could be one of the main means to modulate these processes. Twenty pivotal genetic factors/genes that differentially expressing and being acetylation modified in liver were identified and proposed to regulate the pathogenesis of fatty liver dairies. These proteins were confirmed to be differentially expressing in individual liver tissue, eight of which being validated via immunohistochemistry assay.

**Conclusions:**

This study provided a comprehensive proteome and acetylome profile of fatty liver of dairy cows, and revealed potential important biological processes and essential regulators in the pathogenesis of fatty liver disease. Expectantly, understanding the molecular mechanisms of the pathogenesis of fatty liver disease in dairies, as an animal model of non-alcoholic fatty liver disease (NAFLD) in human beings, which is a clinico-pathologically defined process associated with metabolic syndrome, could inspire and facilitate the development of efficacious therapeutic drugs on NAFLD.

**Supplementary Information:**

The online version contains supplementary material available at 10.1186/s12864-021-07950-2.

## Background

Fatty liver disease is a prevalently occurred metabolic disorder in dairy cows during the transition from dry period to early lactation in commercial farms throughout the world [1, 2], which is caused by obesity before calving and dramatic physiological change and reduction of food intake after calving [3]. Meanwhile, the slowly increasing body lactose consumption easily results in insufficient sugar supply and enhanced fat mobilization in the liver of postpartum dairy cattle, causing immunological stress and/or metabolic challenge. As the central organ of energy and substance metabolism, liver undertakes its mission in maintaining the metabolic balance of carbohydrate, fat and protein in mammals. In addition, the increasing fat mobilization promotes gluconeogenesis and also dramatically induces the production of non-esterified fatty acid (NEFA) in the liver [4, 5]. Although NEFA can be partially re-esterified to triglyceride (TG), a type of very-low-density lipoprotein (VLDL). However, VLDL is hardly transported out of the liver, especially for dairy cattle because of its lack for esterase [3, 6], TG in the liver is prone to be excessively accumulated, causing fatty liver.

It is imperative and pertinent to identify the crucial genetic factors that regulate the pathogenesis of fatty liver disease in dairy cows [2]. The incidence of fatty liver disease at the early lactation not only decreases the milk yield of the coming lactation period, but also limits the serving life because of subsequent health problems of dairy herds [7]. Second, the perinatal disorders remain as prevalent now as they were 20 years ago [1], causing worldwide high culling rate of dairy cows in their early lactation period (within 60 days of lactation), approximately 24 % in USA [8] and 27−30 % in China [9]. Clinical strategies, such as increasing blood calcium levels and using anti-inflammatory drugs had being applied in practice [8], however, could not either completely change the situation or alleviate the contradictions. Thus, identification of crucial genetic factors that regulate the pathogenesis of fatty liver disease in dairy cows would be essential and benefit for the discovery of novel diagnostic or prewarning biomarkers, and also be supportive for the development of safer and more effective therapeutics to prevent and/or treat fatty liver disease in animals. Eventually, these are potentially important constituents contributing to the sustainable development of dairy industry [2].

Recent studies indicate that protein acetylation in mitochondria is suggested to be closely associated with negative energy balance of dairy cattle during their early lactation stage [10, 11]. Although previous proteome profiling studies on ketosis in dairy cattle, another type of commonly occurred metabolic disorder during parturition period, were performed, the underlying pathogenesis of fatty liver disease in dairy cows remains largely unknown [3, 12, 13]. It would be vigorous to reveal the essential pathology and pathogenesis of fatty liver disease by means of updated proteome analysis [14], so as to fundamentally identify the underneath crucial genetic factors/genes regulate the development of fatty liver disease in patients and in animals.

## Results

**Fig. 1:**
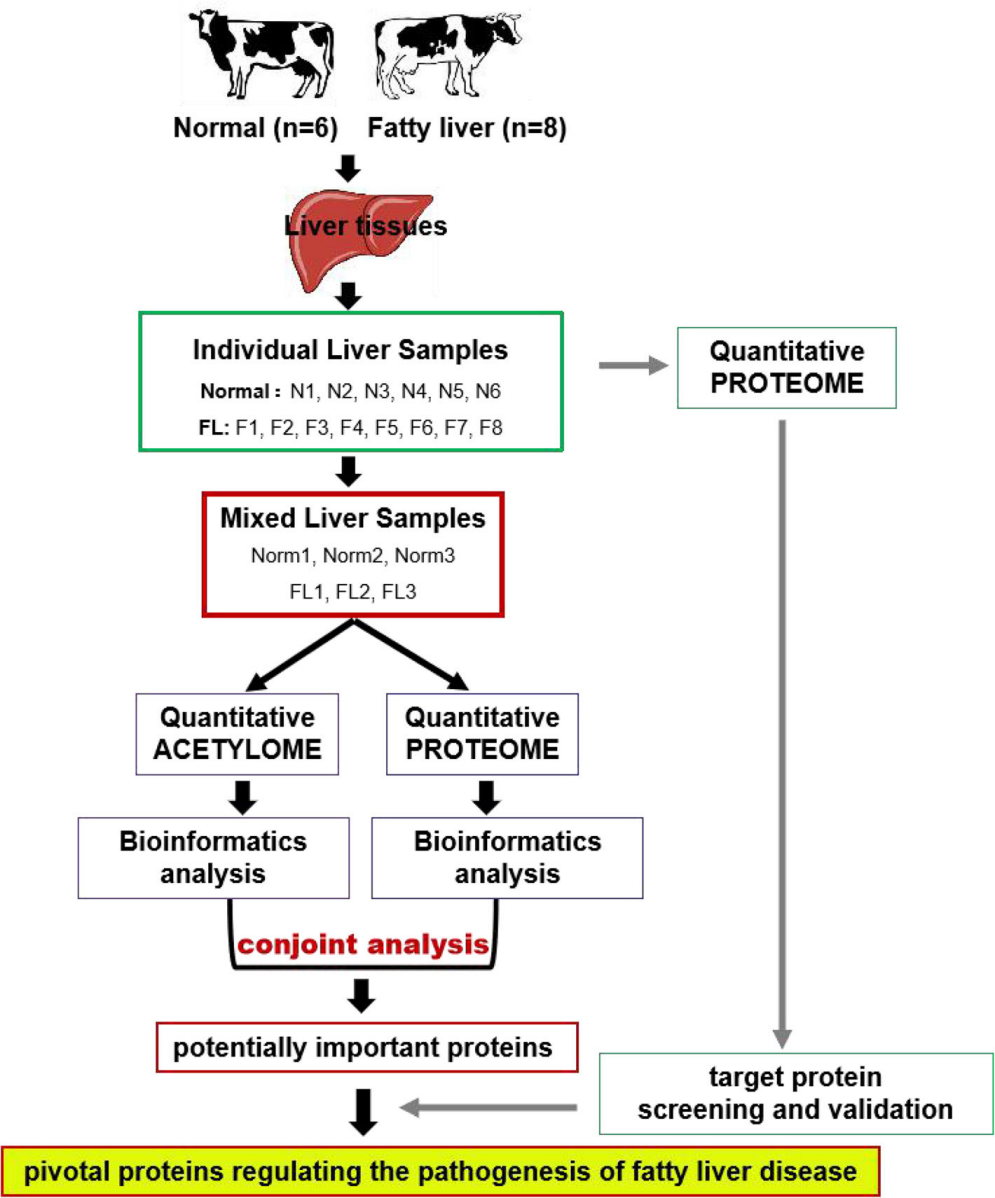
Experimental work-flow. The liver tissue samples of 6 normal cows (Norm) and eight fatty liver cows (FL) were individually applied for proteome analysis. Meanwhile, these samples were equal proportionally and randomly mixed into three biological replicates according to our previous report [11], respectively. Half of each replicate sample was applied for acetylome and/or proteome analysis. The differential expressed and/or acetylated proteins between the fatty liver group and the normal group detected by mass spectrometry were performed bioinformatic analysis, further screened and then validated, so as to identify the pivotal regulatory proteins in the pathogenesis of fatty liver disease

The study was carried out as the work-flow shown in Fig. 1. The postpartum dairy cows were assessed for the occurrence of fatty liver disease by serum biochemical traits examination and then chosen for liver biopsy (Table S1). The biopsied liver samples were detected for the assessment of fat content by oil red O staining, averagely with 6.26 % (*n* = 6) and 86.75 % (*n* = 8) of cells containing lipid droplets, named as normal (Norm) and fatty liver (FL) groups. Both proteome and acetylome sequencing analysis were then performed by using three pairs of biological replicates that were randomly reorganized from 6 individual normal and/or 8 fatty liver tissues [11]. The identified crucial proteins, with significantly different expression levels and lysine acetylation levels, were testified by the expression data obtained from the individually proteome sequencing data. In a word, proteome data obtained through mixed and individual liver sample sequencing, plus the same source of acetylome data, were all contributed for the identification of the pivotal protein candidates controlling the hepatic metabolism, especially for fat deposition in liver. The detailed results were shown step-by-step as follows (Fig. 1).

### Energy metabolism associated DEPs were down-regulated, and immune response activation associated DEPs were up-regulated

The heat map of Pearson’s correlation coefficients showed that biological replicates within group had positive correlation and replicates between Norm and FL groups had negative correlation (Fig. S1A). Both of the distribution range (Fig. S1B) and the length of identified peptide (Fig. S1C) suggest that the preparation of proteome sequenced samples was appropriate, the obtained peptide data was reliable, and the model used in the study was credible.

**Fig. 2:**
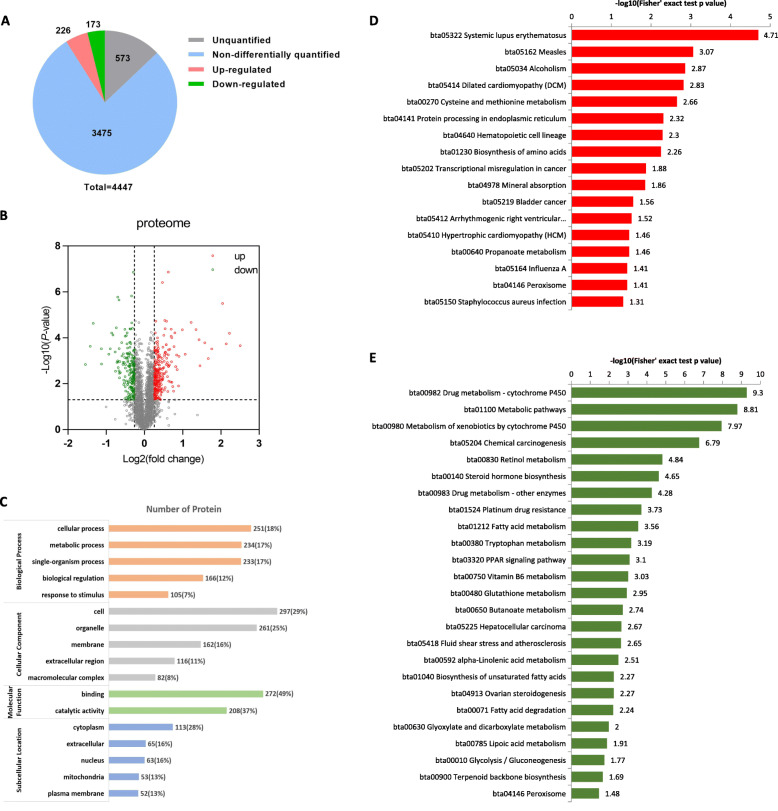
Functional annotation of differentially expressed proteins identified from mix-sampled proteome data. **A** Distribution of the identified proteins by mass spectrometry. Gray: Number of proteins that is not quantified to express abundance; Blue: Number of proteins that are not differentially expressed in disease group comapred to normal group; Red: up-regulated proteins (Fold change > 1.2, *P* < 0.05); Green: down regulated proteins (Fold change < 1/1.2, *P* < 0.05). **B **Volcanic map of the differentially expressed proteins. Red dots: up-regulated proteins (Fold change > 1.2, *P* < 0.05); Green: down-regulated proteins (Fold change < 1/1.2, *P* < 0.05). **C** Gene Ontology (GO, www.ebi.ac.uk/GOA/) functional classification and subcellular location information of differentially expressed proteins. **D** Enriched KEGG (www.kegg.jp/kegg/kegg1.html) pathways by up-regulated proteins. **E** Enriched KEGG pathways by down-regulated proteins

A total of 4,447 proteins were identified, of which 3,874 were quantified. Further, using a *P*-value filter under 0.05 with a difference of 1.2-fold or more, we identified 226 up-regulated and 173 down-regulated proteins in fatty liver group (Fig. 2A and B), respectively.

Functional annotated enrichment analysis of GO and KEGG was performed for all the identified 399 differentially expressed proteins (DEPs). Biological process enrichment revealed that molecular function of DEPs were mainly associated with binding (49 %) and catalytic activity (37 %); and these proteins were mainly distributed in cytoplasm (28 %), nucleus (16 %), extracellular (16 %) and mitochondria (13 %) (Fig. 2C). KEGG enrichment revealed that the up-regulated proteins are mainly involved in pathways associated with immunodeficient diseases and/or related protein processing and biosynthesis (Fig. 2D); the down-regulated proteins are mainly involved in energy metabolism, such as cyclochrome P450 related metabolism, chemical carcinogenesis, stereoid hormone biosynthesis, fatty acid metabolism, tryptophan metabolism and/or PPAR signaling pathway (Fig. 2E).

**Fig. 3:**
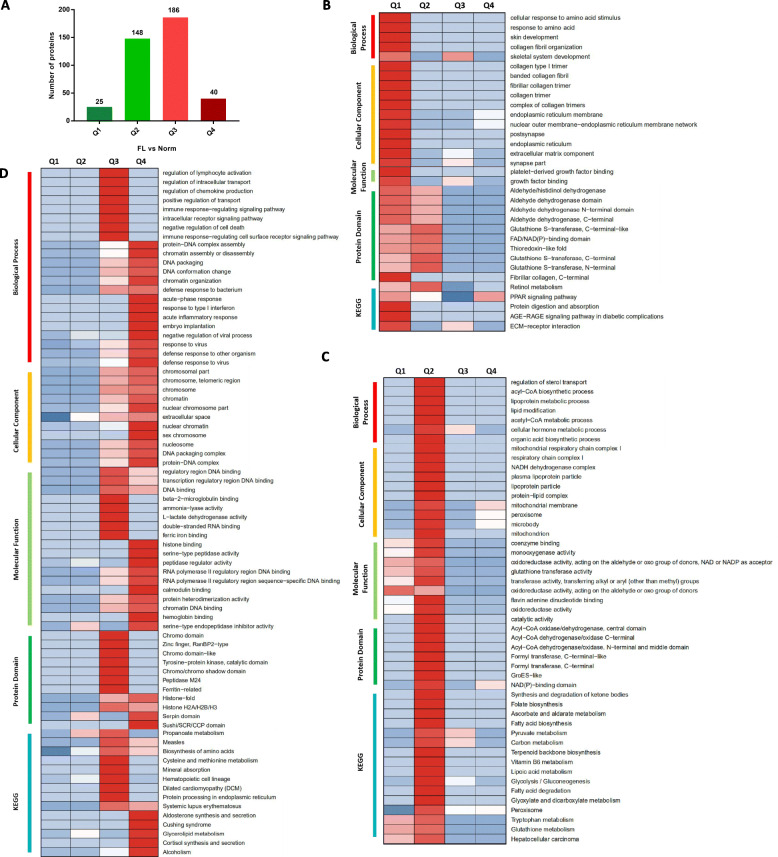
Cluster enrichment analysis of differential expressed proteins identified from proteome data in aspects of biological processes, cellular components, molecular functions, protein domains and KEGG pathways. **A** Distribution of all the identified differentially expressed proteins, being divided them into four categories according to their expression change fold: Q1 (0 < Ratio ≤ 1/1.5), Q2 (1/1.5 < Ratio ≤ 1/1.2), Q3 (1.2 < Ratio ≤ 1.5) and Q4 (Ratio > 1.5). **B** Cluster enrichment analysis of differentially expressed proteins in Q1 class. **C** Cluster enrichment analysis of differentially expressed proteins in Q2 class. **D** Cluster enrichment analysis of differentially expressed proteins in Q3 and Q4 class

**Fig. 4:**
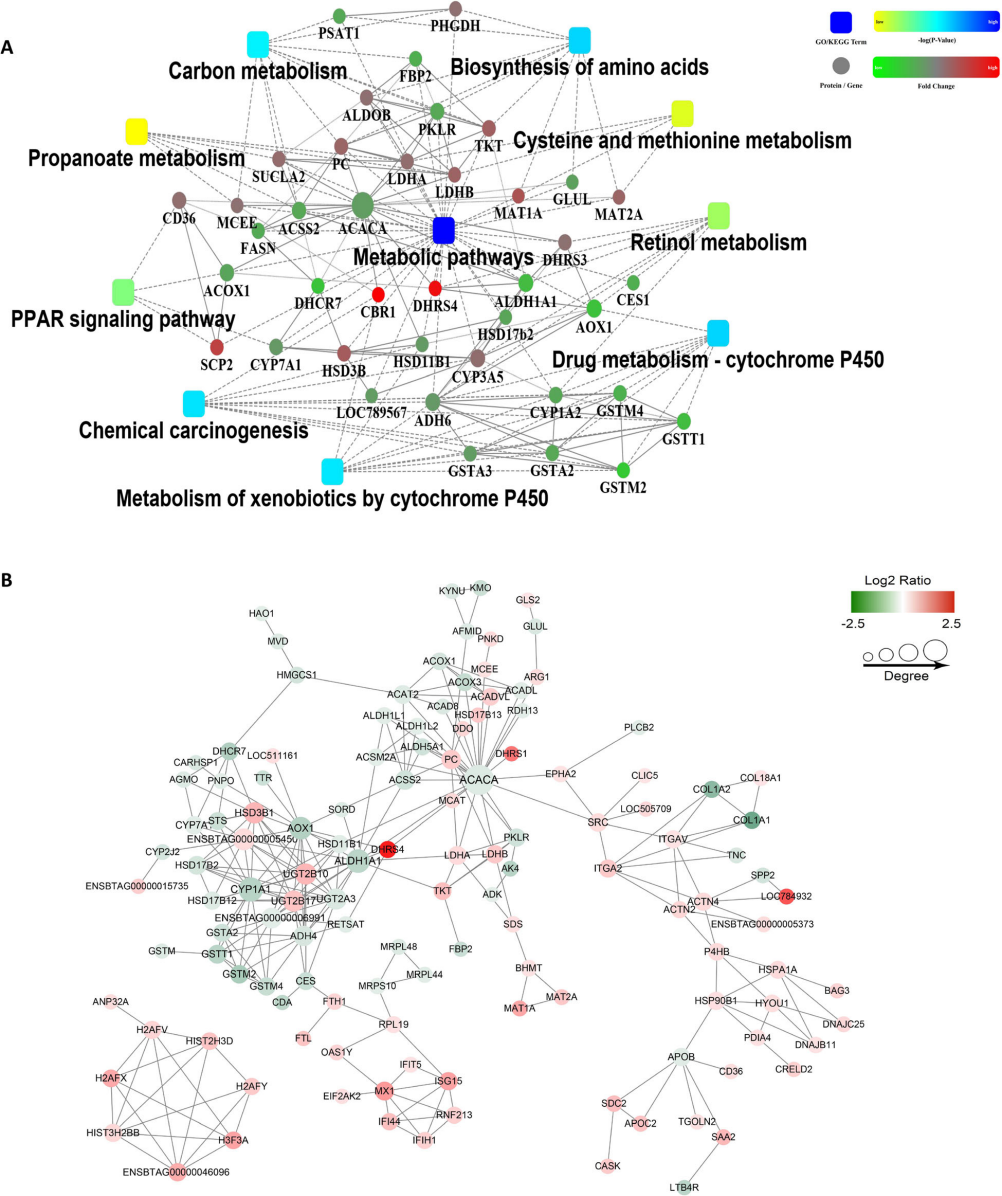
Protein-protein interaction (PPI) analysis of differential expressed proteins identified by mix-sampled proteome data. **A** Interaction networks of all differencially expressed proteins showing significantly enriched pathways and key proteins (http://string-db.org/). Circle size represents the interaction intensity of proteins. Circle color represents the regulation direction, up-regulated (Fold change > 1.2, *P* < 0.05) proteins in red and down-regulated (Fold change < 1/1.2, *P* < 0.05) proteins in green. Square color represents the significance of enriched pathways. **B** protein-protein interaction networks of differencially expressed proteins. Circle size represents the interaction intensity of proteins. Circle color represents the same sense as in **A**

To further elucidate the possible function of these DEPs, they were divided into 4 different categories: Q1 (25 DEPs), Q2 (148 DEPs), Q3 (186 DEPs), and Q4 (40 DEPs) (Fig. 3A). Enrichment analysis of GO, KEGG and protein domains enriched in each Q group was performed. The proteins in Q1 category, with bigger down-regulated fold change, mainly bind to growth factors and participate in biological processes related to retinol metabolism and PPAR signaling pathways (Fig. 3B). Proteins in Q2 category with smaller down-regulated fold change, are mainly involved in a large number of energy metabolism associated pathways that being occurred in mitochondria by modifying their oxidoreductase and/or transferase activities, such as fatty acid metabolism, glycolysis/gluconeogenesis, carbon metabolism, amino acid metabolism, pyruvate metabolism and ketone body synthesis and degradation (Fig. 3C). While, as for the DEPs in Q3 and Q4 category, the enrichment results show many overlapping parts, indicating up-regulated proteins are extensively enriched in DNA assembly and/or degradation, biological processes of immune defense responses, or disease-related pathways (Fig. 3D). These enriched possible biological processes and/or pathways were also identified and confirmed by the interaction network analysis (Fig. 4A).

Amazingly, the enriched distribution of the DEPs got high consistence with that of differentially acetylated proteins (DAPs) identified by acetylome data [11], suggesting there are definitely crucial proteins playing regulatory roles in liver metabolism. Similar KEGG enriched pathways were obtained from the DEPs and DAPs. For example, pyruvate metabolism pathway was both enriched by up-regulated proteins and higher-acetylated proteins. Especially for the down-regulated proteins, most of the enriched pathways were overlapped between proteome and acetylome (Fig. S2A), such as drug metabolism - cytochrome P450, metabolism of xenobiotics by cytochrome P450, chemical carcinogenesis, retinol metabolism, steroid hormone biosynthesis, drug metabolism - other enzymes, platinum drug resistance. It suggested that the acetylation might be one of the major modification means of these important proteins regulating the cellular metabolism in the liver. However, the protein expression level and their acetylated level showed weakly positive correlation coefficient (Fig. S2B). This indicated that the function of acetylated protein might be consistent with the protein itself, but the acetylaion modified direction (higher or lower) and/or regulated level might be weakly correlated with that of the protein itself.

These collective results of enrichment analysis suggest that the pathogenesis of fatty liver disease in dairy cattle was accompanied with energy metabolism suppression (mainly occurred in mitochondria, for example, tricarboxylic acid cycle, TCA cycle) and immune reaction activation. This was again confirmed by protein-protein interaction networks of these DEPs. In the protein network, some rate-limiting or key enzymes (proteins) were indicated as central nodes in the complicated protein-protein network. For example, acetyl-CoA carboxylase 1 (ACACA), cytochrome P450 (CYP1A1), retinal dehydrogenase 1 (ALDH1A1) and aldehyde oxidase 1 (AOX1) displayed 26, 16, 15 and 13 connections with other proteins, respectively (Fig. 4B).

### Validation of the identified DEPs

**Fig. 5:**
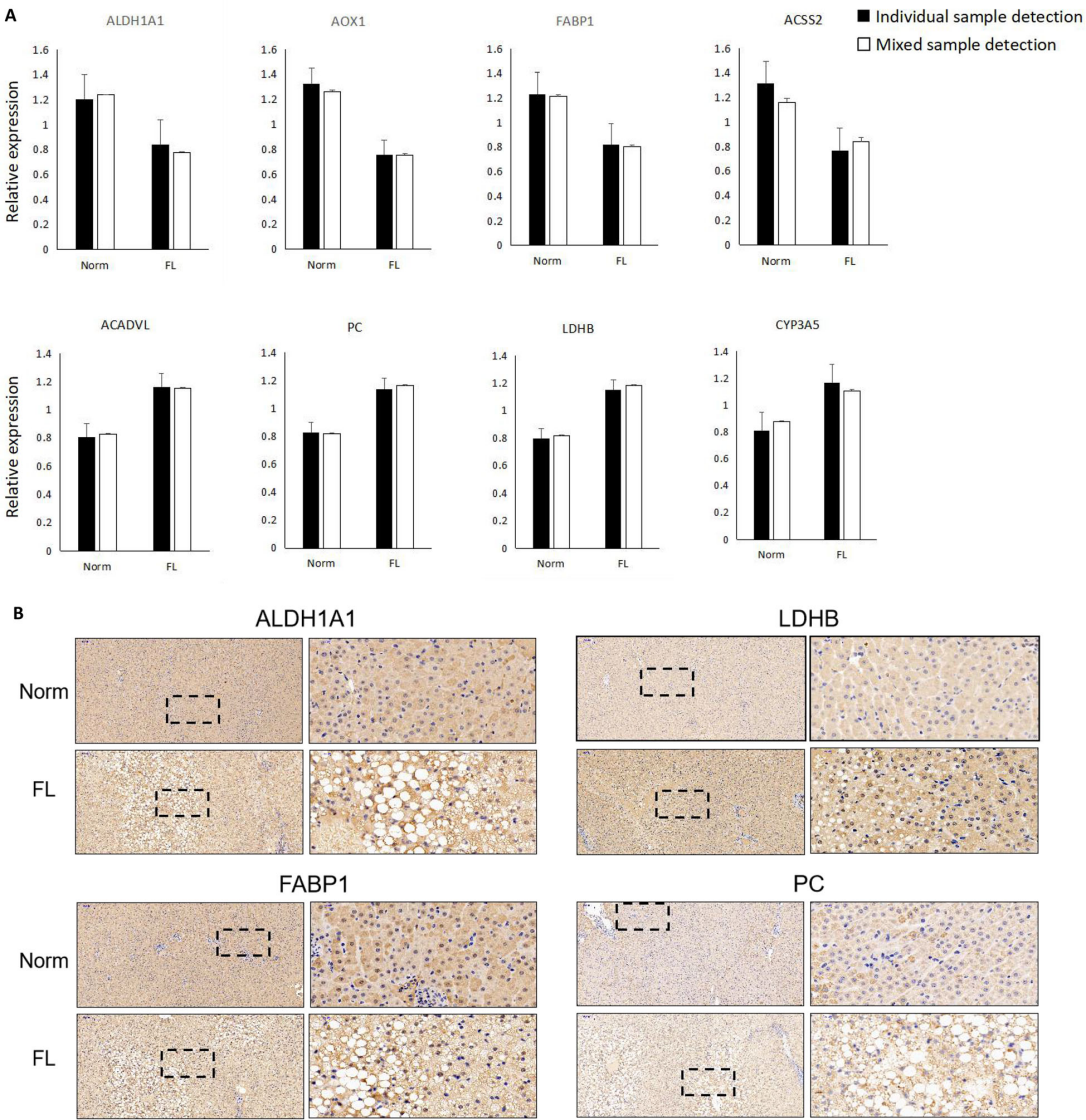
Validation of the identified DEPs. **A** The averaged expression level of randomly selected DEP in individual- (Norm: *n* = 6; FL: *n* = 8) and mix-sampled (Norm: *n* = 3; FL: *n* = 3) liver tissues was shown in parallel. **B** Immunohistochemical staining confirmation of DEPs (ALDH1A1, LDHB, FABP1 and PC) being differentially expressed in normal (Norm) and fatty liver (FL) tissues of Holstein cows. The right panel is a high-powered magnification of the black dashed area in the left panel, with magnification of ×10 (bar = 100 μm) and ×40 (bar = 50 μm), respectively

Another set of proteome data obtained previously by using the same source of liver tissues, but individually sequenced (240 DEPs), were applied in the study, in order to validate the expression levels of proteins in the normal and fatty liver tissues. In the study, eight of the 399 DEPs were randomly selected and compared their expression levels in mixed (*n* = 3 for both normal and fatty liver group) and individual sequenced samples (*n* = 6 for normal group and *n* = 8 for fatty liver group). The results indicated that both 4 down-regulated and 4 up-regulated proteins showed similar expression regulation pattern and good consistency under the two different assessment methods (Fig. 5A), suggesting the reliability of the identified DEPs in the study. Moreover, immunohistochemical staining results of 4 randomly selected proteins also confirmed their expression levels were different in normal and disease liver tissues, indicating consistent protein expression regulation pattern obtained from the two different sets of proteomic data (Fig. 5B). Actually, these liver specific expressed proteins had been confirmed to be important proteins for liver function, such as fatty acid biding protein 1 (FABP1), lactate dehydrogenase B (LDHB) and pyruvate carboxylase (PC).

### Identification of potentially essential proteins regulating the pathogenesis of fatty liver disease

**Fig. 6:**
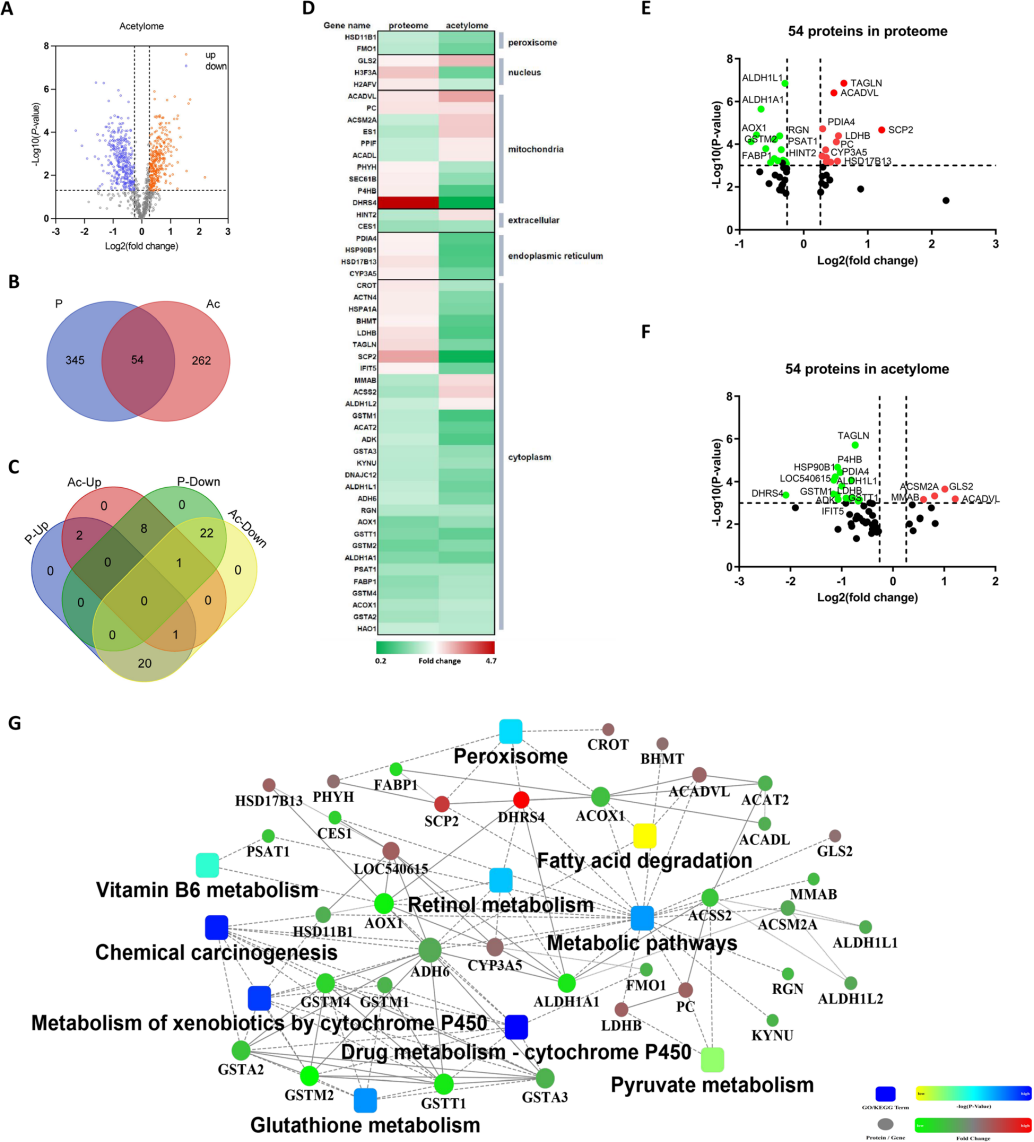
Conjoint analysis of proteome and acetylome data and identification of common regulated/modified proteins (DEPs and DAPs). **A** Volcano plot displaying all quantified differential acetylated proteins in Norm and FL group livers based on the acetylome data. Orange dots represent statistically significant up-regulated proteins (122) and blue dots represent down-regulated proteins (213). **B** Venn diagram displaying 54 overlapping protein that are both significantly regulated in expression abundance and acetylation modified based on proteome and acetylome, respectively. **C** Venn diagram displaying the distribution of the 54 proteins in regulation (P-up and/or P-down) and modification (Ac-up and/or Ac-down) direction. **D** Heat-map showing the subcellular localization of the 54 overlapping proteins according to the Uniprot database annotation. **E** and (**F**)Volcano plot displaying the expression level (**E**) and the acetylation level (**F**) of the 54 overlapping proteins found in proteome and acetylation data, respectively. **G** Protein-protein interaction (PPI) analysis of the overlapping 54 proteins. Circle size represents the interaction intensity of proteins. Circle color represents the regulation direction, up-regulated (Fold change > 1.2, *P* < 0.05) proteins in red and down-regulated (Fold change < 1/1.2, *P* < 0.05) proteins in green. Square color represents the significance of enriched pathways

Since lysine acetylation is an important and wide spread protein modification for diverse proteins involved in energy metabolism, the identified DEPs in the study were jointly analyzed and intersected with the acetylome data obtained by using the same source of liver tissues. The acetylome data revealed that 307 lysine sites on 122 proteins higher-acetylated (fold-change > 1.2, *P* < 0.05) and 358 lysine sites on 213 proteins lower-acetylated (fold-change < 1/1.2, *P* < 0.05) in fatty liver tissues, compared to the normal liver tissues (Fig. 6A). The MS/MS spectra of representative acetylated proteins at specific Kac sites were shown as Fig. S3. Therefore, 54 proteins were identified as overlapping proteins (Fig. 6B) with different regulation patterns, such as 23 down-down proteins (meaning down-regulated in proteome data and lower-acetylated in acetylome data), 3 up-up proteins, 21 up-down proteins and 9 down-up proteins (Fig. 6C). Subcellular localization analysis of these 54 overlapping proteins indicated they were mainly localized in the mitochondria and/or cytoplasm (Fig. 6D). Some of the proteins were shown extremely significant differences in both proteome and acetylome data (Fig. 6E F), such as LDHB, very long-chain specific acyl-CoA dehydrogenase (ACADVL), and aldehyde dehydrogenase 1 family member L1 (ALDH1L1). In addition, protein-protein interaction network analysis of these 54 DEPs showed 100 interactions and 52 nodes were mapped to the protein interaction database (Fig. 6G), allowing us to understand the underneath biological processes and important proteins (nodes) regulating the homeostasis of liver metabolism. Especially, metabolic pathways associated with cytochrome P450, chemical carcinogenesis and pathways associated with amino acid metabolism were all again significantly enriched (Fig. 4A).

### Validation of the overlapping DEPs and identification of pivotal protein candidates regulating the pathogenesis of fatty liver disease

**Fig. 7:**
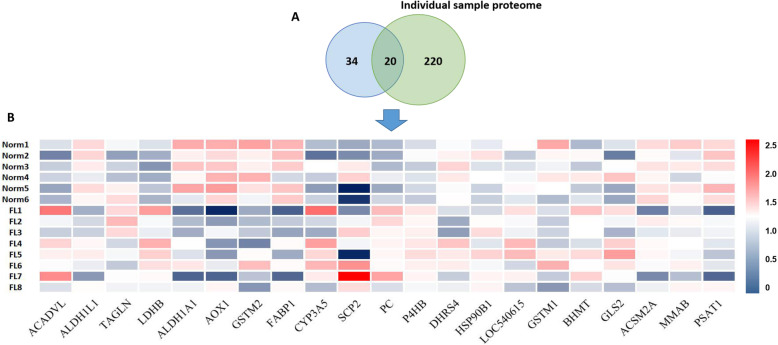
Expression abundance testification of the 54 proteins using the individual sampled proteome data. **A** Venn diagram displaying 20 overlapping proteins between the 54 (34 + 20) proteins and the 240 (220 + 20) DEPs identified from individual proteome data. **B** Heat-map showing the expression profile of the 20 overlapping proteins in individual liver sample based on their proteome data, 6 normal liver (Norm, *n* = 6) and 8 fatty liver (FL, *n* = 8). Red represents up-regulated fold change and green represents down-regulated fold change

The 54 overlapping proteins that presumed to be essential regulator in the pathogenesis of fatty liver disease were again intersected with the 240 DEPs identified by individual proteome data. Therefore, 20 proteins were further filtered out, being considered as the pivotal protein candidates in regulating the pathogenesis of fatty liver disease (Fig. 7A). In a another word, the expression level of the 20 differentially acetylation modified proteins was validated to have significantly different expression abundance in normal and fatty liver tissues, especially for ALDH1A1, AOX1, PC and FABP1 (Fig. 7B). These proteins were proposed to be pivotal biomarkers of fatty liver disease in dairy cattle (Fig. 5A and B).

## Discussion

Fatty liver disease frequently occurs in dairy cows, a clinico-pathologically defined nonalcoholic fatty liver disease (NAFLD), resulting in a high culling rate of dairy cows during their perinatal period, because of limitations of milk production performance and attenuation of reproduction performance in the subsequent serving life. It is estimated that 40–60 % of high-yielding dairy cows (daily milk yield > 35 kg) develop moderate to severe fatty liver disease within 2 weeks after calving [5, 15]. Moreover, the perinatal fatty liver disease remain as prevalent now as they were 20 years ago [1]. The biology underlying the transition to lactation of dairy cattle was considered as the “final frontier” of our understanding the dairy cattle [1, 2]. Rather than attempting to define the transition state by using transcriptome or metabolome, the purpose of this study was to focus on the proteome and its acetylatome of fatty liver as an emerging aspect of transition cow biology, because protein is the ultimate realization agent of the life.

### Mitochondrial dysfunction and inflammation activation were the important biological processes that accompanied with the pathogenesis of fatty liver disease

**Fig. 8:**
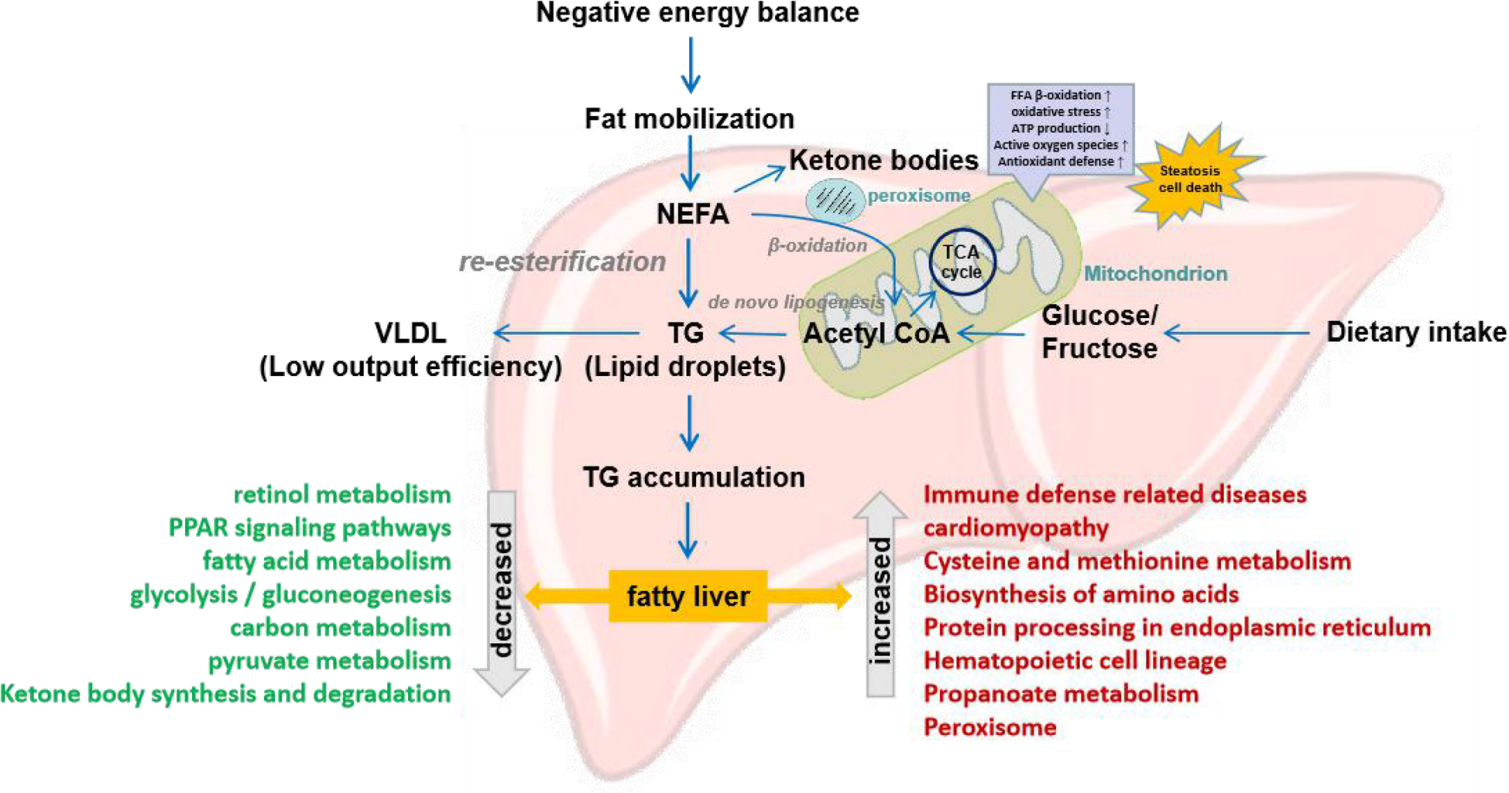
The proposed schematic diagram of significant metabolic changes occurred in the pathogenesis of fatty liver disease in dairy cows. During the perinatal period, the metabolism of dairy cattle is in a state of negative energy balance, promoting the mobilization of fat in the liver. Enhanced fat mobilization promotes a sharp increase in non-esterified fatty acids (NEFA) in the liver. These NEFAs are more inclined to involved into re-esterification and therefore synthesis of triglycerides (TG) and/or production of ketone bodies, but not β-oxidation, because of motochrondrial dysfunction and inflammation causing the increased (red) and/or decreased (green) metabolism processes. The excessive accumulation of TG in the liver would lead to fatty liver

Proteome-wide analysis revealed that the pathogenesis of fatty liver disease in dairy cattle was accompanied with energy metabolism suppression and immune response activation. Majority of DEPs were enriched in the biological processes that closely associated with mitochrondrial functions, such as mitochrondrial respiratory chain complexes, fatty acid metabolism, glycolysis/gluconeogenesis, carbon metabolism, amino acid metabolism, pyruvate metabolism and ketone body synthesis and degradation (Figs. 2D and E and 3B C). Especially, many down-regulated DEPs were found to be involved in energy metabolism occurred in mitochondria (such as tricarboxylic acid cycle/krebs cycle) by modifying their oxidase/dehydrogenase and/or transferase activities (Figs. 2E and 3B C). It was presumed that mitochondrial dysfunction was one of the underlying reason for the onset of fatty liver disease in dairy cattle (Fig. 8) [11].

Mitochondria are crucial for energy metabolism processes in a cell, including the production of more than 90 % of cellular ATP, β-oxidation, apoptosis, cell-cycle progression, proliferation and aging, and their dysfunction has been implicated in a wide range of metabolic diseases [16]. The role of mitochondrial dysfunction was already well-established in NAFLD and non-alcoholic steatohepatitis (NASH), with structural mitochondrial abnormalities, a reduction in mitochondrial respiratory chain activity and overwhelmed β-oxidation (oxidative stress) induced by excessive reactive oxygen species (ROS) production because of increased free fatty acid (FFA) load, therefore resulting in subsequent activation of inflammatory pathways (Fig. 8) [16, 17]. Hepatic lipid accumulation may be initiated by repression of mitochondrial fatty acid oxidation, which consequently causes the imbalance between lipid synthesis and lipid expenditure in liver. Associations between mitochondrial injuries and NAFLD development had been confirmed, mitochondrial dysfunction can inhibit mitochondrial β-oxidation and thereby cause fatty liver and increase lipid toxic metabolites, which might in turn exert adverse effects on mitochondrial function, resulting in a vicious cycle [18–20]. Lipotoxicity and/or oxidative stress caused by fatty acids can result in the swelling of mitochondria (mitochondrial ultrastructural damage), activity reduction of enzymes in mitochondria (impaired respiratory chain function), dysfunction of mitochondria in hepatocytes and eventually apoptosis of hepatocytes. The metabolic disorder caused by mitochondria in hepatocytes is an important cause of liver metabolic disorder and abnormal deposition of fat [16, 17, 19]. One of a recent study on early lactation Holstein-Friesian cows indicated that mitochondrial function was impaired during early lactation in pasture-fed cows, but not in total mixed rotations-fed cows [10]. Interestingly, acetylome analysis also revealed that the TCA cycle occurred in mitochondria was significantly inhibited and the immunoregulatory pathways were activated in the fatty liver tissues of dairy cows [11]. Our findings suggest that mitochondrial dysfunction might play a critical role in the development of fatty liver disease in dairy cattle, and mitochondria could be a target to improve liver function. In addition, it is worthy to mention that increasing experimental evidence points to mitochondrial alterations and/or dysfunction contribute to tumorigenesis and the development of drug resistance, suggesting that mitochondrial targeting might be an effective strategy for chemotherapy [21].

As for the up-regulated DEPs identified by proteome analysis, most of them were significantly enriched into pathways either associated with DNA stability and/or transcription activity or immune defense responses (Figs. 2D and 3D). The conversion of free fatty acid (FFA) to hepatic TG may serve as a protective measure to prevent direct hepatic lipotoxicity. However, excessive hepatic FFA supply can contribute to inflammation, with elevated hepatic expression of inflammatory cytokines and/or adipokines, which had been endorsed by murine models of NAFLD [17, 22]. It was proposed that the abnormality in lipid and lipoprotein metabolism accompanied by chronic inflammation is the central pathway for the development of NAFLD [23]. Actually, inflammation activation was considered as an emerging aspect of transition cow biology [1], studies had clearly shown that essentially all cows would experience systemic subacute inflammation in the several days after parturition, even in the absence of disease, with increased haptoglobin and globulin, exaggerated cytokine response and reduced levels of liver hydrolase paraoxonase [8, 24].

In current study, crucial pathways closely associated with mitochondrial function and/or immune response were identified to play essential regulatory roles in the development of fatty liver disease in dairy cows (Fig. 8), such as metabolic pathways, cyclochrome P450 related metabolism, amino acid metabolism and/or biosynthesis, retinol metabolism and PPAR signaling pathway (Figs. 2D and E and 4A). The possible important dysregulated proteins causing the abnormal metabolism in liver were indicated by protein-protein interaction networks, such as ACACA, CYP1A1, ALDH1A1 and AOX1, displaying vigorous connections with other proteins (Fig. 4B).

### Protein acetylation was the vitally important manner regulating the pathogenesis of fatty liver disease

Comparison analysis of the proteome and acetylome data indicated that DEPs obtained from proteome were highly consistent with the annotation of DAPs identified from acetylome, with similar GO enrichment results (Fig. 2 C, Fig. S2A) [11]. Intriguingly, our previous acetylome data indicated that most of the proteins with higher acetylation level were significantly enriched in energy and amino acid metabolic pathways, while proteins with lower acetylation level were significantly enriched in immune response and/or disease-related pathways [11]. This was exactly opposite to the enrichment results obtained from expression level down-regulated proteins (Figs. 2E and 3C and D) and up-regulated proteins (Figs. 2D and 3D). This suggests that lysine acetylation might be a vitally important post-transcriptional modification manner on these metabolism modulating proteins. In another word, the effect of the acetylation on the functional modification of the target protein could play critical roles in the development of fatty liver disease in dairy cows.

Moreover, the enriched pathways from proteome analysis got high consistence with that of acetylome analysis [11], with plenty of pathways and/or biological processes commonly enriched, such as pyruvate metabolism, cyclochrome P450 related metabolism, stereoid hormone biosynthesis, retino metabolism and glycolysis/gluconegesis (Fig. S2A), suggesting these acetylated proteins play essential regulatory roles in the pathogenesis of fatty liver disease via participating in these mitochondrial metabolism pathways. Amazingly, a considerable portion of DEPs are located in mitochondria, and their acetylation levels have been significantly modified, such as ACADVL, ALDH1L1, LDHB (Fig. 6E F) [11]. The acetylation of these proteins were confirmed to be involved in different aspects of energy metabolism in mitochondria, such as TCA cycle, β-oxidation (fatty acid oxidation), lipid metabolism, therefore being associated with NAFLD [16, 25–29]. Protein acetylation has a crucial role in energy metabolism. Changes in cellular nutrient availability or energy status can induce global changes in mitochondrial protein acetylation [16, 30]. For example, insulin inhibits β-oxidation in the mitochondria through modifying the protein acetylation [10, 31–33], resulting in the accumulation of lipids in hepatocytes. Lysine acetylation, as a common post-transcriptional modification of proteins, is vitally important in both immunological and metabolic pathways and regulates the balance between energy storage and expenditure [32]. Additionally, acetyl-CoA, as the core product of TCA cycle in mitochondria, is an indicator of cellular energy status [16, 32]. Protein acetylation could be a convergence point for carbohydrate, amino acid and fat metabolism, clearly emerging as a common post-transcriptional modification in mitochondria and fluctuating the metabolic enzyme activity. Mitochondrial protein acetylation is sensitive to metabolic perturbations, sensing the energy/nutrient deprivation or excess and even ethanol or environmental exposure [10, 16, 20, 30, 31, 34].

Histone acetyltransferses (HATs) and histone deacetylase (HDACs) mediate acetylation and deacetylation of histone proteins and transcription factors, as a modulator to modify the acetylation level of target proteins. For example, mammalian sirtuins (SIRTs), NAD-dependent histone deacetylase such as SIRT1 and SIRT3, were involved in oxidative stress and lipid metabolism regulation [33], and had been proposed as a reliable biomarker and/or therapeutic target for fatty liver disease [10, 19, 33, 35, 36]. Especially in dairy cows, a significant increase in protein lysine acetylation was found in pasture-fed Holstein-Friesian dairy cows during their early lactation, accounting for impaired hepatic mitochrondrial function in this period [10].

### Pivotal proteins/genetic factors, such as AOX1 and ALDH1A1, was identified as important regulators in the pathogenesis of fatty liver disease

Since both proteome and acetylome data indicated that mitochondrial energy metabolism suppression and inflammation activation (disease defense) were shown as the underlying reasons for pathogenesis of fatty live disease in dairy cattle, it would be remarkable to identify the corresponding functional proteins. Conjoint analysis of DEPs from proteome and DAPs from acetylome identify 54 proteins that were significantly expressed in fatty liver tissues and their acetylation levels were also widely modified (Fig. 6). Interestingly, protein-protein interaction network analysis of these 54 proteins got similar biological pathway enrichment with that based on all DEPs from proteome analysis, such as metabolic pathways, metabolism associated with cytochrome P450, chemical carcinogenesis, retinol metabolism and pathways associated with amino acid metabolism (Figs. 4 and 6G A). Moreover, the 54 proteins kept and recognized most of the core proteins with abundant interactions with other proteins in the DEPs, such as CYP1A1, AOX1, ALDH1A1, DHRS4 (dehydrogenase/reductase SDR family member 4), GSTMs (glutathione S-transferase mu), etc. (Figures 4B and 6G). More importantly, 20 proteins were further confirmed to have significantly different expression level in normal liver and fatty liver tissues (Figs. 5 and 7). These identified proteins were supposed to be pivotal proteins/genetic factors that regulating the pathogenesis of fatty liver disease in dairy cattle, not only for their significant different expression abundance but also significant different acetylation modification levels.

Some of the 20 proteins had previously been reported to closely associate with the onset of NAFLD and/or its related biological processes. For example, AOX1 is a cytoplasmic enzyme that is highly expressed in the liver and plays a key role in the metabolism of drugs containing aromatic heterocyclic substituents. AOX1 can produce reactive oxygen species, which can promote cell damage and fibrosis. Fatty liver pathogenesis is associated with elevated liver AOX1 [37], while adiponectin can inhibit the expression of AOX1 by activating PPARɑ, therefore enhancing the lipid oxidation, attenuating the inflammation reaction and alleviating the liver injuries [22]. Another example is ALDH1A1, mainly expressed in the cytoplasm, playing important regulatory roles in the development of fatty liver. Both AOX1 and ALDH1A1 are involved in the retinol metabolic pathway. It is well-known that retinoic acid is a key regulator of glucose and lipid metabolism in the liver and adipose tissue. Liver diseases, especially those that cause fibrosis and cirrhosis, are related to impaired vitamin A (retinol) homeostasis and/or vitamin A deficiency [37]. It has been proved that ALDH1A1 can catalyze the oxidation of retinol to retinoic acid. ALDH1A1 knockout mice are resistant to obesity induced by high-fat diet, indicating that ALDH1A1 may be a candidate gene for obesity treatment [38].

In this study, some previously confirmed to be functional important in liver were also identified to be significantly regulated. For example, FABP1 and GSTM2 were down-regulated, PC and LDHB were up-regulated. FABP1, as a transport of fatty acids, can inhibit the cellular lipotoxicity caused from FA and regulate the synthesis and distribution of lipids in cells [39, 40]. The down-regulation of FABP1 in the liver will lead to the impairment of FA uptake and suppression of the production of VLDL, therefore increasing the accumulation of liver lipids [41, 42]. GSTM2, mu subtypes of glutathione-S-transferase, has enzymatic function of eliminating toxic and harmful factors (free radicals, peroxides and electrophilic groups) *in vitro* and *in vivo*, playing a role in protecting cells and regulating cell growth [43, 44]. Lactate dehydrogenase (LDH) and pyruvate carboxylase (PC) are important enzyme catalyzing the metabolism in mitochondria. Liver-specific PC knockout mice developed exacerbated oxidative stress and elevated liver inflammation, along with suppressed gluconeogenesis [45], indicating the specific necessity of PC for maintaining oxidation, biosynthesis, and pathways distal to TCA cycle.

These core proteins were recognized based on liver-specific proteome and acetylome analysis, as well as validation by individual proteome data, because of their close associations with immune response and/or energy metabolism, especially with metabolic pathways occurred in mitochondria. These proteins were identified as important candidate genetic factors that regulate the pathogenesis of fatty liver disease in dairy cattle, could potentially be developed into accurate and reproducible biomarkers to diagnose and/or pre-warn the risk of fatty liver dairies and also therapeutic targets for the disease treatment. Of course, it would be of our next interest to elucidate the detailed molecular functions in the pathology and pathogenesis of fatty liver disease in dairy cattle.

Additionally, NAFLD is a clinico-pathologically defined process associated with metabolic syndrome and fundamentally pin-pointed to the pathogenesis of lipid metabolism [26], causing obesity, type II diabetes, liver disease (such as steatohepatitis, fibrosis), threatening the health of humans and animals. Although the research on NAFLD biomarkers has advanced in the last two decades, there are still no reliable biomarkers for the diagnosis or the staging of the disease (NAFLD vs. NASH) [46, 47]. NAFLD usually occurred accompanying with increased plasma insulin and fatty acid concentration, elevated fasting aminotransferase (AST/ALT) and/or TG level, and also abnormal lipid accumulation in the liver [25, 48]. Moreover, another of the most important risk factors is histological evidence of hepatic inflammation [49] caused by acute inflammation and subacute inflammation [1, 15]. Meanwhile, NAFLD is a metabolic disease closely related with the acetylation of histones and non-histones [50, 51]. Dairy cows with fatty liver disease is a typical (non-progressive) NAFLD animal model, with similar fundamental metabolic disorder syndrome. The identification of pivotal proteins in dairy cattle fatty liver disease would be beneficial to inspire and refresh our understanding of the pathology and pathogenesis of NAFLD.

## Conclusions

Fatty liver disease prevalently occurs in postpartum dairy cattle, causing a worldwide high culling rate of dairies after calving. There is an urgent need to develop accurate and reproducible biomarkers to predict or diagnose the progression of the disease. Pivotal genetic factors/genes that are proposed to regulate the pathogenesis of fatty liver disease in dairy cattle are identified in the study by focusing on the liver-specific proteomics and acetylome analysis. Some of them were confirmed to be significantly differential expressing in individual fatty livers. The energy metabolism suppression mainly caused by mitochondrial dysfunction and inflammation activation are implicated to be remarkable processes in the development of fatty liver disease in dairy cattle. Protein acetylation might play crucial modifying roles through participating in different aspects of these significant processes/pathways. Persistent elucidation of the molecular mechanisms of these proteins/genes in the development of fatty liver disease in dairy cattle would be greatly beneficial to develop novel effective therapeutic targets to prevent and/or treat the disease, contributing to the sustainable development of dairy industry. The study also provides a novel road-map to an understanding of the intriguing portion of the “final frontier” of dairy cow biology. Additionally, since the fundamental metabolic syndrome of fatty liver disease in cattle is pretty similar with that in NAFLD patients, understanding the molecular mechanisms of the pathogenesis of fatty liver disease in dairies will further enhance our understanding of NAFLD in human beings and promoting the efficacious drug research and development.

## Methods

### Ethics statement

All animal experiments were carried out according to the Regulations for the Administration of Affairs Concerning Experimental Animals published by the Ministry of Science and Technology, China (2004), and were approved by the Shandong Agricultural University Animal Care and Use Committee (approval number, SDAUA-2017-044).

### Liver tissue biopsy of dairy cattle

Dairy cattle after parturition around 1–2 weeks was examined for the serum biochemical traits and thereby targeted for liver biopsis according to the detailed procedures [11], so as to assess the fat deposition in liver and thereby diagnose the cows with fatty liver disease. The detailed information of biopsied dairy cattle was listed in Table S1. Eventually, six normal liver samples (Norm group, labeled as N1-N6, averagely with percentage of cells contained lipid drops 6.26 ± 5.23 %) and eight severe fatty liver samples (FL group, labeled as F1-F8, with percentage of cells contained lipid drops 86.75 %±4.83 %) were randomly regrouped in an equal proportion for proteome analysis, named as Norm1, Norm2, Norm3, and FL1, FL2, FL3, respectively. Meanwhile, all the 14 samples were performed individual proteome sequencing detection. The work flow of the study was shown in the Fig. 1.

### TMT labeling, LC-MS/MS analysis, database searching and data quality control

The tryptic peptide segments of each replicate sample were then treated and labeled according to the manufacturer’s protocol of TMT kit and then fractionated by high pH reverse HPLC via the column of Agilent 300Extend C18 column (5 µ m particle size, 4.6 mm inner diameter, 250 mm long) according to the standard LC-MS/MS analysis protocol (PTM-BioLab, Hangzhou, China). The peptides were subjected to NSI source followed by tandem mass spectrometry (MS/MS) in Q ExactiveTM Plus (Thermo) coupled online to the UPLC. The electrospray voltage applied was 2.0 kV. The m/z scan range was 350 to 1800 for full scan, and intact peptides were detected in the Orbitrap at a resolution of 70,000. Peptides were then selected for MS/MS using NCE setting as 28 and the fragments were detected in the Orbitrap at a resolution of 17,500. A data-dependent procedure that alternated between one MS scan followed by 20 MS/MS scans with 15.0 s dynamic exclusion. Automatic gain control (AGC) was set at 5E4. Fixed first mass was set as 100 m/z.The resulting MS/MS data were processed using Maxquant search engine (v.1.5.2.8). Tandem mass spectra were searched against UniProt *Bos taurus* (24,215) database concatenated with reverse decoy database. Trypsin/P was specified as cleavage enzyme allowing up to 2 missing cleavages. The mass tolerance for precursor ions was set as 20 ppm in First search and 5 ppm in Main search, and the mass tolerance for fragment ions was set as 0.02 Da. Carbamidomethyl on Cys was specified as fixed modification and oxidation on Met was specified as variable modifications. FDR was adjusted to < 1 % and minimum score for peptides was set > 40.

### Identification of differentially expressed protein peptides

The differentially expressed proteins (DEPs) were identified when meeting the criteria of statistically significant (*P* < 0.05) fold change ≥ 1.2 and/or ≤ 1/1.2. The DEPs were also divided into 4 categories according to their fold change: Q1 (0 < Ratio ≤ 1/1.5), Q2 (1/1.5 < Ratio ≤ 1/1.2), Q3 (1.2 < Ratio ≤ 1.5), and Q4 (Ratio > 1.5).

### Bioinformatics analysis

Gene Ontology (GO, www.ebi.ac.uk/GOA/) and Kyoto Encyclopedia of Genes and Genomes (KEGG, www.kegg.jp/kegg/kegg1.html) enrichment analysis were carried out by using the online service tool [52–54]. Protein-protein interaction analysis was then assessed by using the software Wolfpsort to annotate the subcellular localization of the protein, STRING database (version 10.5; confidence score ≥ 0.7) to search the protein-protein interactions and molecular complex detection (MCODE) to analyse and visualize the network. The clustering relationship is visualized by the heat map drawn by Heatmap. 2 program in gplots R package.

### Validation of the differentially expressed proteins

The identified DEPs were validated by applying our previous proteome data that were obtained via individual sample sequencing (Fig. 1). The individual sequenced proteome data were obtained from 6 normal individual liver tissue samples and 8 individual severe fatty liver tissue samples based on the same TMT technology (PTM-BioLab, Hangzhou, China). Thereafter, 240 DEPs were captured by using the same criteria (fold change > 1.2 or fold change < 1 / 1.2 and *P* < 0.05). Thus, the two sets of DEPs were compared and intersected to obtain the overlapping proteins.

The expression abundance of each DEP were partially selected to validate the expression fold change in fatty liver tissues compared to that in normal tissues, via parallelly showing their expression levels in two different sets of proteome data. Several liver-specifically proteins that was previously confirmed to be functional important in liver were included as positive controls, such as FABP1, LDHB and PC.

### Cojoint analysis of proteome and acetylome data

The DEPs identified by the proteome data was compared with that of the differentially acetylated proteins (DAPs) identified by the acetylome data based on the same tissue samples, therefore the overlapped proteins were filtered out, being presumed to be potentially important proteins that are closely related to liver lipid metabolism, because of their significant different expression abundance and acetylation modification levels. Then, their subcellular localization, functional annotation and protein interaction network analysis were carried out.

### Identification of pivotal proteins controlling the pathogenesis of fatty liver disease

In order to further filter the pivotal proteins regulating the pathogenesis of fatty liver disease in dairy cattle, the overlapped DEPs (obtained by intersecting DEPs from proteome and acetylatome data) were again intersected with all the set of DEPs identified from individual proteome data. The pivotal proteins were identified by calculating the correlation coefficient of expression abundance of each protein in mixed and individual liver samples.

### Statistical significance

The significant differentially expressed proteins were defined using *t*-test, as with 1.2-fold change threshold and *P* value < 0.05. The Pearson correlation coefficient was used to assess the repeatability of biological replicates. As for the functional annotation/enrichment analysis, the enrichment significance is defined by *P* value < 0.05, and further filtered through converting the *P* value (obtained from Fisher’s exact test) into negative logarithm (- log10).

### Immunohistochemical analysis

Liver tissues biopsied from postpartum dairy cows were fixed in 4 % paraformaldehyde and embedded in paraffin. The tissue sections were then subjected to standard immunohistochemical staining and photomicrography [55]. Rabbit primary and HRP-labeled goat anti-rabbit secondary antibodies were used in a diluted working solution (FABP1, 1: 400; ALDH1A1, 1: 200; LDHB, 1: 500; PC, 1: 1000. Sanying Ltd. Co., Wuhan, China).

## Supplementary Information

**Additional file 1:** Fig. S1. Sample quality and data reliability assessment. Fig. S2. Crosstalk analysis between proteome and acetylome. Fig. S3. MS/MS spectra of GOT2 K90 (A), MDH2 K328 (B), HADHA K516 (C), HMGCS2 K350 (D) acetylation.

**Additional file 2: Table S1.** Production information of normal (Norm) and fatty liver dairy cows that were liver biopsied and their serum biochemical parameters^1^

## Data Availability

The data used to support the findings of this study are available from the corresponding author upon request.

## References

[1] Bradford BJ, Yuan K, Farney JK, Mamedova LK, Carpenter AJ (2015). Invited review: Inflammation during the transition to lactation: new adventures with an old flame. J Dairy Sci.

[2] Shi KR, Li RR, Xu ZJ, Zhang Q (2020). Identification of crucial genetic factors, such as PPARγ, that regulate the pathogenesis of fatty liver disease in dairy cows is imperative for the sustainable development of dairy industry. Animals (Basel).

[3] Bobe G, Young JW, Beitz DC (2004). Invited review: pathology, etiology, prevention, and treatment of fatty liver in dairy cows. J Dairy Sci.

[4] Reid IM, Rowlands GJ, Dew AM, Collins RA, Roberts CJ, Manston R (1983). The relationship between post-parturient fatty liver and blood composition in dairy cows. J Agr Sci-Cambridge.

[5] Shi KR, Niu FG, Zhang Q, Ning C, Yue SJ, Hu CZ (2020). Identification of whole-genome significant single nucleotide polymorphisms in candidate genes associated with serum biochemical traits in Chinese Holstein cattle. Front Genet.

[6] Katoh N (2002). Relevance of apolipoproteins in the development of fatty liver and fatty liver-related peripartum diseases in dairy cows. J Vet Med Sci.

[7] Carvalho MR, Penagaricano F, Santos JEP, DeVries TJ, McBride BW, Ribeiro ES (2019). Long-term effects of postpartum clinical disease on milk production, reproduction, and culling of dairy cows. J Dairy Sci.

[8] Carpenter AJ, Ylioja CM, Vargas CF, Mamedova LK, Mendonca LG, Coetzee JF (2016). Hot topic: Early postpartum treatment of commercial dairy cows with nonsteroidal antiinflammatory drugs increases whole-lactation milk yield. J Dairy Sci.

[9] Li SL, Huang WM, Tian YJ, Cao ZJ. Energy metabolism and its regulation of perinatal dairy cows. Res Prog Anim Nutr 2012;170–6.

[10] Garcia-Roche M, Casal A, Mattiauda DA, Ceriani M, Jasinsky A, Mastrogiovanni M (2019). Impaired hepatic mitochondrial function during early lactation in dairy cows: Association with protein lysine acetylation. PLoS One.

[11] Zhang LT, Hu CZ, Zhang X, Zhang Q, Yan ZG, Wei QQ (2020). Protein acetylation in mitochondria plays critical functions in the pathogenesis of fatty liver disease. BMC Genom.

[12] Niu L, Geyer PE, Wewer Albrechtsen NJ, Gluud LL, Santos A, Doll S (2019). Plasma proteome profiling discovers novel proteins associated with non-alcoholic fatty liver disease. Mol Syst Biol.

[13] Xu C, Wang Z (2008). Comparative proteomic analysis of livers from ketotic cows. Vet Res Commun.

[14] Altelaar AF, Munoz J, Heck AJ (2013). Next-generation proteomics: towards an integrative view of proteome dynamics. Nat Rev Genet.

[15] Starke A, Schmidt S, Haudum A, Scholbach T, Wohlsein P, Beyerbach M, Rehage J (2011). Evaluation of portal blood flow using transcutaneous and intraoperative Doppler ultrasonography in dairy cows with fatty liver. J Dairy Sci.

[16] Anderson KA, Hirschey MD (2012). Mitochondrial protein acetylation regulates metabolism. Essays Biochem.

[17] Dowman JK, Tomlinson JW, Newsome PN (2010). Pathogenesis of non-alcoholic fatty liver disease. QJM.

[18] van der HanKolk JH, Gross JJ, Gerber V, Bruckmaier RM (2017). Disturbed bovine mitochondrial lipid metabolism: a review. Vet Q.

[19] Grattagliano I, Montezinho LP, Oliveira PJ, Fruhbeck G, Ambrosi JG, Montecucco F (2019). Targeting mitochondria to oppose the progression of nonalcoholic fatty liver disease. Biochem Pharmacol.

[20] He XW, Gao J, Hou H, Qi ZD, Chen HM, Zhang XX (2019). Inhibition of mitochondrial fatty acid oxidation contributes to development of nonalcoholic fatty liver disease induced by environmental cadmium exposure. Environ Sci Technol.

[21] Ghosh P, Vidal C, Dey S, Zhang L (2020). Mitochondria targeting as an effective strategy for cancer therapy. Int J Mol Sci.

[22] Polyzos SA, Perakakis N, Mantzoros CS (2019). Fatty liver in lipodystrophy: a review with a focus on therapeutic perspectives of adiponectin and/or leptin replacement. Metabolism.

[23] Fon TK, Rozman D (2011). Nonalcoholic Fatty liver disease: focus on lipoprotein and lipid deregulation. J Lipids.

[24] Nightingale CR, Sellers MD, Ballou MA (2015). Elevated plasma haptoglobin concentrations following parturition are associated with elevated leukocyte responses and decreased subsequent reproductive efficiency in multiparous Holstein dairy cows. Vet Immunol Immunopathol.

[25] Demir M, Lang S, Steffen HM (2015). Nonalcoholic fatty liver disease - current status and future directions. J Dig Dis.

[26] Tiniakos DG, Vos MB, Brunt EM (2010). Nonalcoholic fatty liver disease: pathology and pathogenesis. Annu Rev Pathol.

[27] Day CP (2011). Non-alcoholic fatty liver disease: a massive problem. Clin Med (Lond).

[28] Duvnjak M, Lerotic I, Barsic N, Tomasic V, Virovic Jukic L, Velagic V (2007). Pathogenesis and management issues for non-alcoholic fatty liver disease. World J Gastroenterol.

[29] Zhang J, Cai BL, Ma MT, Luo W, Zhang ZP, Zhang XQ, Nie QH (2020). ALDH1A1 inhibits chicken preadipocytes’ proliferation and differentiation via the PPARγ pathway in vitro and in vivo. Int J Mol Sci.

[30] Schwer B, Eckersdorff M, Li Y, Silva JC, Fermin D, Kurtev MV (2009). Calorie restriction alters mitochondrial protein acetylation. Aging Cell.

[31] Guo L, Guo YY, Li BY, Peng WQ, Chang XX, Gao X (2019). Enhanced acetylation of ATP-citrate lyase promotes the progression of nonalcoholic fatty liver disease. J Biol Chem.

[32] Iyer A, Fairlie DP, Brown L (2012). Lysine acetylation in obesity, diabetes and metabolic disease. Immunol Cell Biol.

[33] Zhang YQ, Zhou FY, Bai MY, Liu Y, Zhang LL, Zhu Q (2019). The pivotal role of protein acetylation in linking glucose and fatty acid metabolism to beta-cell function. Cell Death Dis.

[34] Thapa D, Wu K, Stoner MW, Xie B, Zhang M, Manning JR (2018). The protein acetylase GCN5L1 modulates hepatic fatty acid oxidation activity via acetylation of the mitochondrial beta-oxidation enzyme HADHA. J Biol Chem.

[35] Guo L, Zhou SR, Wei XB, Liu Y, Chang XX, Liu Y (2016). Acetylation of mitochondrial trifunctional protein ɑ-subunit enhances its stability to promote fatty acid oxidation and is increased in nonalcoholic fatty liver disease. Mol Cell Biol.

[36] Li Y, Zou SP, Ding HY, Hao N, Huang YY, Tang JS (2020). Low expression of Sirtuin 1 in the dairy cows with mild fatty liver alters hepatic lipid metabolism. Animals (Basel).

[37] Saeed A, Dullaart RPF, Schreuder TCMA, Blokzijl H, Faber KN (2017). Disturbed Vitamin A Metabolism in Non-Alcoholic Fatty Liver Disease (NAFLD). Nutrients.

[38] Haenisch M, Treuting PM, Brabb T, Goldstein AS, Berkseth K, Amory JK (2018). Pharmacological inhibition of ALDH1A enzymes suppresses weight gain in a mouse model of diet-induced obesity. Obes Res Clin Pract.

[39] Peng XE, Wu YL, Lu QQ, Hu ZJ, Lin X (2012). Two genetic variants in FABP1 and susceptibility to non-alcohol fatty liver disease in a Chinese population. Gene.

[40] Rodriguez SL, Arias NMB, Scaglia N, Lockhart LJF, Franchini GR, Storch J (2017). FABP1 knockdown in human enterocytes impairs proliferation and alters lipid metabolism. Biochim Biophys Acta Mol Cell Biol Lipids.

[41] Guzman C, Benet M, Pisonero-Vaquero S, Moya M, Garcia-Mediavilla MV, Martinez-Chantar ML (2013). The human liver fatty acid binding protein (FABP1) gene is activated by FOXA1 and PPARalpha; and repressed by C/EBPalpha: Implications in FABP1 down-regulation in nonalcoholic fatty liver disease. Biochim Biophys Acta.

[42] Wang YJ, Tang KQ, Zhang W, Guo WL, Wang YN, Zan LS, Yang WC (2019). Fatty acid-binding protein 1 increases steer fat deposition by facilitating the synthesis and secretion of triacylglycerol in liver. PLoS One.

[43] Chanas SA, Jiang Q, McMahon M, McWalter GK, McLellan LI, Elcombe CR, Henderson CJ, Roland Wolf C, Moffat GJ, Itoh K, Yamamoto M, Hayes JD (2002). Loss of the Nrf2 transcription factor causes a marked reduction in constitutive and inducible expression of the glutathione S-transferase Gsta1, Gsta2, Gstm1, Gstm2, Gstm3 and Gstm4 genes in the livers of male and female mice. Biochem J.

[44] Seidegard J, Pero RW, Miller DG, Beattie EJ (1986). A glutathione transferase in human leukocytes as a marker for the susceptibility to lung cancer. Carcinogenesis.

[45] Cappel DA, Deja S, Duarte JAG, Kucejova B, Inigo M, Fletcher JA (2019). Pyruvate-carboxylase-mediated anaplerosis promotes antioxidant capacity by sustaining tca cycle and redox metabolism in liver. Cell Metab.

[46] Araujo LCC, Feitosa KB, Murata GM, Furigo IC, Teixeira SA, Lucena CF (2018). Uncaria tomentosa improves insulin sensitivity and inflammation in experimental NAFLD. Sci Rep.

[47] Younossi ZM, Loomba R, Rinella ME, Bugianesi E, Marchesini G, Neuschwander-Tetri BA (2018). Current and future therapeutic regimens for nonalcoholic fatty liver disease and nonalcoholic steatohepatitis. Hepatology.

[48] Nasr P, Ignatova S, Kechagias S, Ekstedt M (2018). Natural history of nonalcoholic fatty liver disease: a prospective follow-up study with serial biopsies. Hepatol Commun.

[49] Bellentani S (2017). The epidemiology of non-alcoholic fatty liver disease. Liver Int.

[50] Chung S, Hwang JT, Park JH, Choi HK (2019). Free fatty acid-induced histone acetyltransferase activity accelerates lipid accumulation in HepG2 cells. Nutr Res Pract.

[51] Ferramosca AD, Giacomo M, Zara V (2017). Antioxidant dietary approach in treatment of fatty liver: new insight and updates. World J Gastroenterol.

[52] Kanehisa M, Goto S (2000). KEGG: Kyoto encyclopedia of genes and genomes. Nucleic Acids Res.

[53] Kanehisa M (2019). Toward understanding the origin and evolution of cellular organisms. Protein Sci.

[54] Kanehisa M, Furumichi M, Sato Y, Ishiguro-Watanabe M, Tanabe M. KEGG: integrating viruses and cellular organisms. Nucleic Acids Res. 2021;49:D545–D551. 10.1093/nar/gkaa970 PMC7779016 33125081

[55] Cao QQ, Li HH, Liu X, Yan ZG, Zhao M, Xu ZJ (2019). MiR-24-3p regulates cell proliferation and milk protein synthesis of mammary epithelial cells through menin in dairy cows. J Cell Physiol.

