# Supplementary material for: Proteome analysis identified proteins associated with mitochondrial function and inflammation activation crucially regulating the pathogenesis of fatty liver disease
Source: BMC Genomics. 2021 Sep 4;22:640. doi: 10.1186/s12864-021-07950-2 (PMC8418032; doi:10.1186/s12864-021-07950-2)
Supplement: Supplementary file 1 — Additional file 1: Fig. S1. Sample quality and data reliability assessment. Fig. S2. Crosstalk analysis between proteome and acetylome. Fig. S3. MS/MS spectra of GOT2 K90 (A), MDH2 K328 (B), HADHA K516 (C), HMGCS2 K350 (D) acetylation. [file 12864_2021_7950_MOESM1_ESM.docx]

**Proteome analysis identified proteins associated with mitochondrial function and inflammation activation crucially regulating the pathogenesis of fatty liver disease**

**Authors**

Letian Zhang^#^, Tingjun Liu^#^, Chengzhang Hu, Xuan Zhang, Qin Zhang , Kerong Shi ^*^

**Affiliations**

College of Animal Science and Technology, Shandong Agricultural University, Shandong Key Laboratory of Animal Bioengineering and Disease Prevention, Taian, Shandong, 271018, P. R. China.

**#** These authors contribute equally.

***Address for Correspondence**

Kerong Shi, College of Animal Science and Technology, Shandong Agricultural University, Shandong Key Laboratory of Animal Bioengineering and Disease Prevention, No. 61 Daizong Street, Taian, Shandong, 271018, P. R. China. Work Telephone: 86-538-8246501. Email: [krshi@sdau.edu.cn](mailto:krshi@sdau.edu.cn).


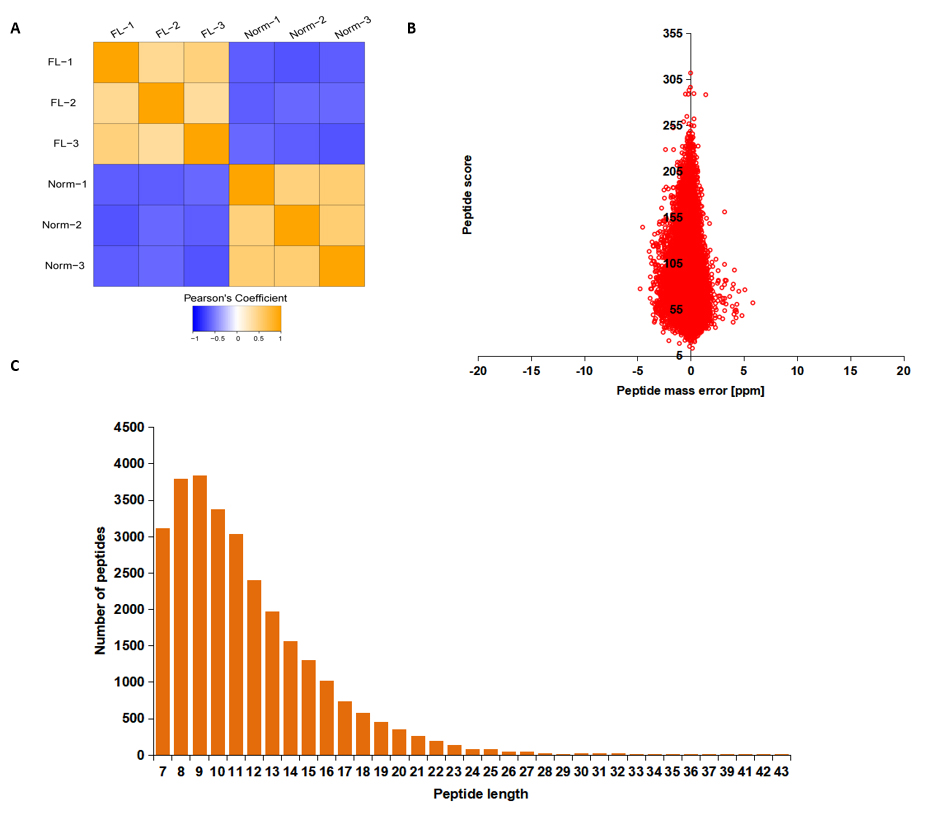


**Fig. S1. Sample quality and data reliability assessment.** (A) Pearson's correlation coefficients between the Norm and FL group samples indicate qualified sample model. (B) Mass error distribution between - 5ppm and 5ppm of all peptides identified by Mass spectrometry, suggesting qualified peptide captured in the study. (C) The length distribution of the identified peptide segments.


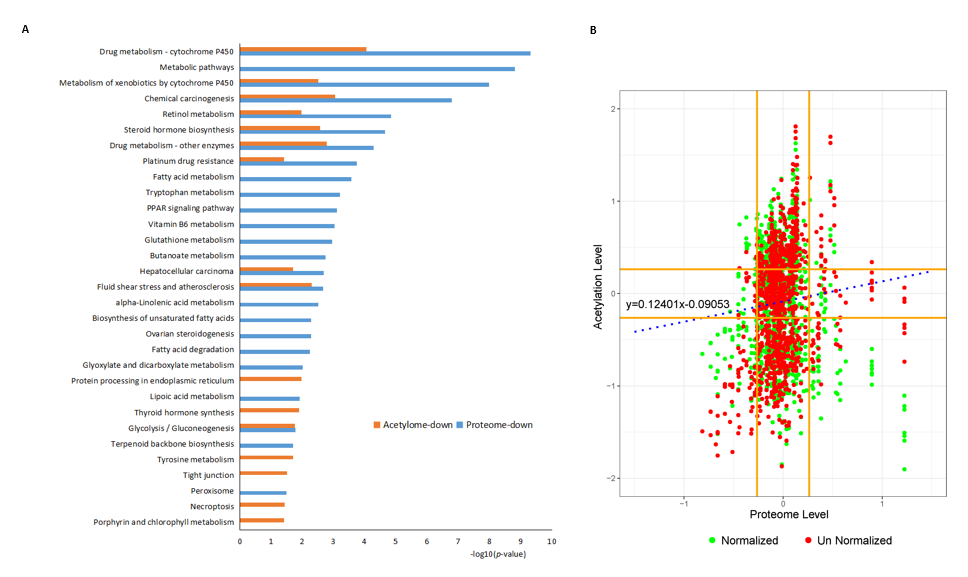


**Fig.S2. Crosstalk analysis between proteome and acetylome.** (A)The overlapping pathways between proteomic downregulated proteins and acetylated downregulated proteins. Orange: significant pathways enriched for proteins down-regulated by acetylation; Blue: significant pathways enriched for down-regulated proteins identified by the proteome. (B) Linear fit all scatter points to draw blue fit line. Abscissa: protein differential expression in proteomic quantitative comparison group (log2). Ordinate: the difference expression of protein sites in acetylated modified quantitative comparison group (log2). Green is the modified group normalized according to proteome, red is not normalized. The four orange lines were the difference thresholds of acetylated group and proteome group.

**A B**


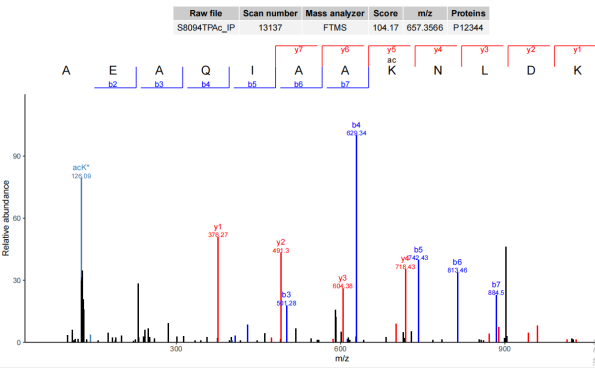

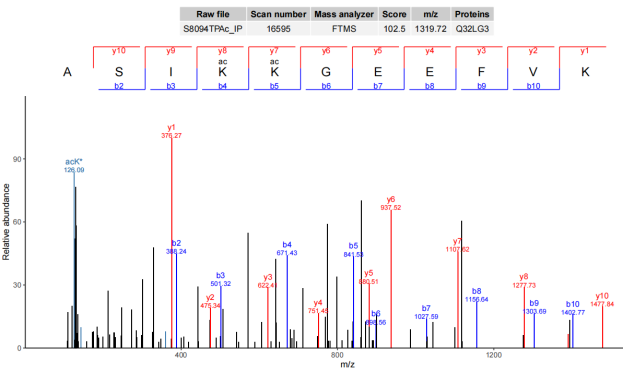


**C D**


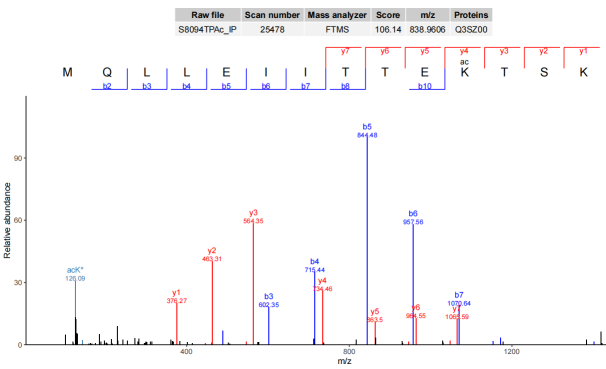

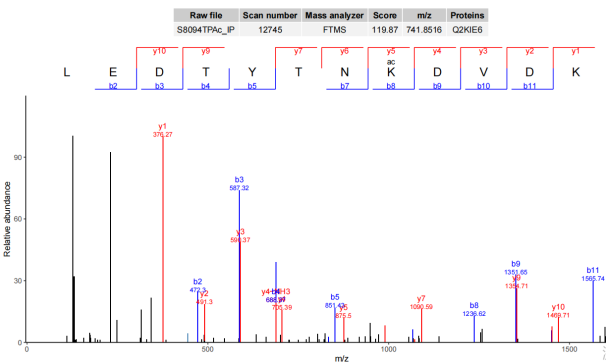


**Fig. S3. MS/MS spectra of GOT2 K90 (A), MDH2 K328 (B), HADHA K516 (C), HMGCS2 K350 (D) acetylation.** The b ion refers to the N-terminal parts of the peptide, and the y ion refers to the C-terminal parts of the peptide.
