# Supplementary material for: Proteome analysis identified proteins associated with mitochondrial function and inflammation activation crucially regulating the pathogenesis of fatty liver disease
Source: BMC Genomics. 2021 Sep 4;22:640. doi: 10.1186/s12864-021-07950-2 (PMC8418032; doi:10.1186/s12864-021-07950-2)
Supplement: Supplementary file 2 — Additional file 2: Table S1. Production information of normal (Norm) and fatty liver dairy cows that were liver biopsied and their serum biochemical parameters1 [file 12864_2021_7950_MOESM2_ESM.docx]

**Proteome analysis identified proteins associated with mitochondrial function and inflammation activation crucially regulating the pathogenesis of fatty liver disease**

**Authors**

Letian Zhang^#^, Tingjun Liu^#^, Chengzhang Hu, Xuan Zhang, Qin Zhang, Kerong Shi^*^

**Affiliations**

College of Animal Science and Technology, Shandong Agricultural University, Shandong Key Laboratory of Animal Bioengineering and Disease Prevention, Taian, Shandong, 271018, P. R. China.

**#** These authors contribute equally.

***Address for Correspondence**

Kerong Shi, College of Animal Science and Technology, Shandong Agricultural University, Shandong Key Laboratory of Animal Bioengineering and Disease Prevention, No. 61 Daizong Street, Taian, Shandong, 271018, P. R. China. Work Telephone: 86-538-8246501. Email: [krshi@sdau.edu.cn](mailto:krshi@sdau.edu.cn).

**Table S1 Production information of normal (Norm) and fatty liver dairy cows that were liver biopsied and their serum biochemical parameters ^1^**

| **Items** | **Norm ^2^ (n=6)** | **FL ^2^ (n=8)** | ***P*-value** |
| --- | --- | --- | --- |
| Fat percentage in liver (%) **^3^** | 6.26±5.23 | 86.75±4.83 | 2.59E-11 |
| Parity No. | 1.17±0.41 | 1.38±0.74 | 0.55 |
| Dry matter intake (kg/d) | 20.6±2.3 | 19.4±3.0 | 0.22 |
| Day in milk (day) | 7±2 | 7±2 | 1.00 |
| Body weight (kg) | 650±53 | 668±45 | 0.34 |
| Milk yield (kg/d) | 26.2±1.9 | 25.3±3.2 | 0.67 |
| INS (pg/ml) | 171.37±26.46 | 268.34±183.83 | 0.45 |
| NEFA(mmol/L) | 1.60±0.88 | 2.78±1.74 | 0.16 |
| BHB (mmol/L) | 78.55±10.64 | 73.54±28.04 | 0.79 |
| GLU (mmol/L) | 3.05±0.64 | 1.93±0.72 | 0.011 |
| AST (IU/L) | 92.83±50.94 | 178.75±116.39 | 0.12 |
| TP (g/L) | 72.17±13.39 | 63.98±7.98 | 0.18 |
| ALB (g/L) | 28.88±3.76 | 29.48±3.44 | 0.76 |
| SUN (μmol/L) | 3.09±0.73 | 4.41±2.39 | 0.22 |
| UA (mol/L) | 36.83±7.63 | 31.63±10.65 | 0.33 |
| TG (μmol/L) | 0.13±0.03 | 0.10±0.03 | 0.19 |
| TCHO (μmol/L) | 2.01±0.64 | 1.63±0.29 | 0.16 |

^1^ INS, insulin; NEFA, non-esterified fatty acid; BHB, β-hydroxybutyric acid; GLU, glucose ; AST, aspartate aminotransferase; TP, total protein; ALB, serum albumin; SUN, serum urea nitrogen; UA, urea acid; TG, triglyceride; TCHO, total cholesterol.

^2^ Norm, normal cows; FL, fatty liver cows.

^3^ It shows the averaged percentage of cells containing lipid droplets in liver tissue, which indicated by Oil Red staining.
